# Supplementary material for: Evaluation of a Single Determination of Gluten Immunogenic Peptides in Urine from Unaware Celiac Patients to Monitor Gluten-Free Diet Adherence
Source: Nutrients. 2023 Mar 2;15(5):1259. doi: 10.3390/nu15051259 (PMC10004805; doi:10.3390/nu15051259)

Supplementary figure S1. Distribution of atrophy along the small bowel of patients with a positive or negative urinary gluten peptides test

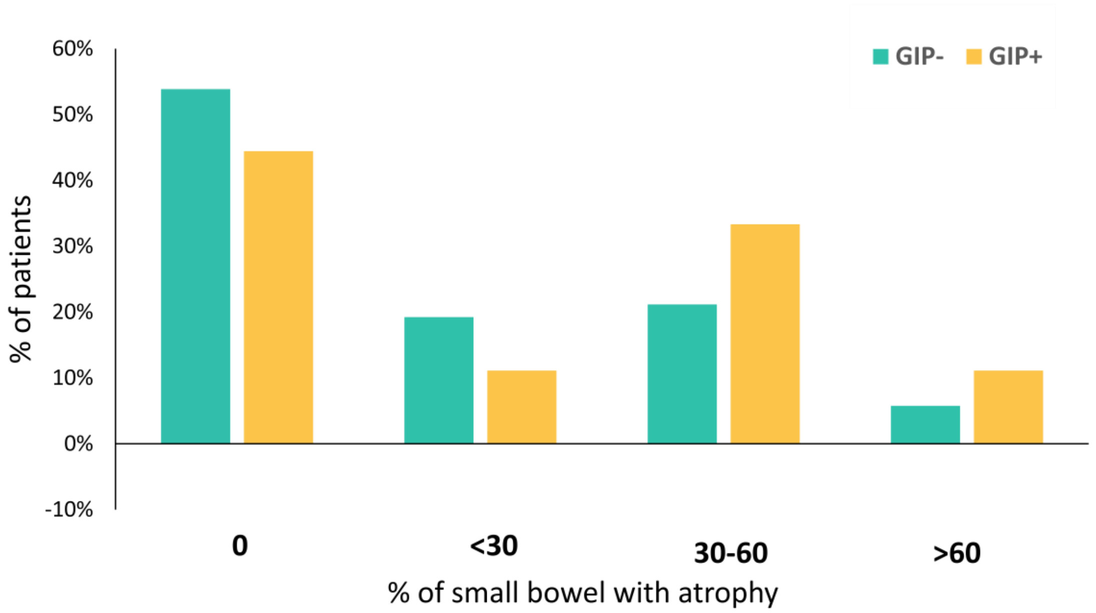

Supplement: Supplementary file 1 [file nutrients-15-01259-s001.zip › nutrients-2198463-supplementary.pdf]
